# Waiting time for bariatric and metabolic surgery in the Brazilian public health system and comorbidities: what is the impact?

**DOI:** 10.1590/0102-672020260000025e1954

**Published:** 2026-08-31

**Authors:** Fernando de BARROS, Leonardo Halamy PEREIRA, Ana Beatriz FONSECA, Breno Gonçalves da SILVA, Daniel Alejandro ENCALADA, Thais Leiros Kleinsorgen MOTTA, Eduardo Pinho BRAGA

**Affiliations:** 1 Universidade Federal Fluminense, Department of General and Specialized Surgery – Niterói (RJ), Brazil.; 2 Universidade Federal Fluminense, Medical Sciences Unit – Niterói (RJ), Brazil.; 3 Universidade Federal Fluminense, Statistics Service – Niterói (RJ), Brazil.; 4 Hospital São Francisco na Providência de Deus, Bariatric Surgery Service – Rio de Janeiro (RJ), Brazil.

**Keywords:** Bariatric surgery, Metabolic syndrome, Public health, Demography, Obesity, Cirurgia bariátrica, Síndrome metabólica, Saúde pública, Demografia, Obesidade

## Abstract

**Background:**

Obesity is a multifactorial disease with a high prevalence that leads to several comorbidities, posing significant challenges for healthcare systems. Bariatric and metabolic surgery (BMS) has been established as the most effective treatment for patients with obesity; however, in Brazil, limited access remains a critical barrier.

**Aims:**

This study aimed to evaluate the clinical, demographic, and metabolic characteristics of patients with obesity undergoing BMS in the Brazilian Unified Health System and to analyze the relationship between waiting time and comorbidities.

**Methods:**

A retrospective cohort study was conducted involving 1,000 patients with obesity who underwent treatment between July 2022 and June 2024. Clinical, anthropometric, and laboratory variables were analyzed using regression analysis and statistical tests to assess the association between waiting time and comorbidities.

**Results:**

A significant correlation was found between prolonged waiting time and an increased number of comorbidities (R²=0.686; p<0.001). Furthermore, the number of comorbidities explained 60% of the variability in waiting time (R²=0.600; p<0.001), with a mean increase of 1.92 years for each additional comorbidity (95%CI 1.82–2.02). Patients on the waiting list for more than 10 years had higher rates of hypertension, type 2 diabetes, and dyslipidemia. Waiting time also had an impact on some metabolic syndrome parameters, including glycated hemoglobin (Hb1Ac) (r=+680, p=0.031), low-density lipoprotein (LDL) (r=+640, p=0.044), and total cholesterol (r=+830, p=0.008).

**Conclusions:**

Prolonged waiting time for bariatric and metabolic surgery is associated with an increased burden of metabolic comorbidities and their consequences.

## ARTICLE HIGHLIGHTS

Early intervention is crucial for achieving better outcomes in most diseases. Similarly, early bariatric and metabolic surgery in patients with obesity can lead to improved cardiovascular outcomes, including reduced mortality and preventable morbidity.

A significant correlation was found between prolonged waiting time and an increased number of comorbidities (R²=0.686; p<0.001). Furthermore, the number of comorbidities explained 60% of the variability (R²=0.600; p<0.001), with a mean increase of 1.92 years for each additional comorbidity (95%CI 1.82–2.02).

Prolonged waiting time for bariatric and metabolic surgery is associated with an increased burden of metabolic comorbidities and their consequences.

## Summary

**CENTRAL MESSAGE:**

Bariatric and metabolic surgery (BMS) has been shown to be effective and safe in ameliorated comorbidities. The medical literature supports the notion that early intervention is crucial for achieving better outcomes in most diseases. Similarly, early BMS in patients with obesity can lead to improved cardiovascular outcomes, including reduced mortality and a lower incidence of cardiovascular events. BMS has also been shown to have renoprotective effects, potentially helping to prevent renal failure and significantly reducing the risk of progression from chronic kidney disease to kidney failure. Furthermore, BMS is associated with a 49.2% reduction in the hazard rate of death compared to usual care, with a median increase in life expectancy of 6.1 years.

**PERSPECTIVES:**

Prolonged waiting time for bariatric and metabolic surgery was associated with an increased number of comorbidities and their complications, particularly hypertension, type 2 diabetes, and dyslipidemia. Similarly, laboratory parameters associated with metabolic syndrome, such as glycated hemoglobin (HbA1c), low density protein (LDL), and total cholesterol, also worsened significantly with delayed bariatric surgery.

## VISUAL ABSTRACT

**Figure:**
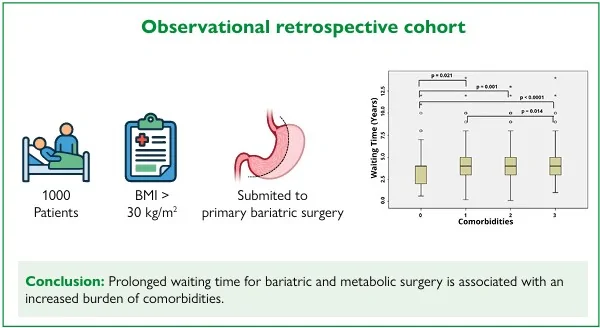

## RESUMO

**Racional:**

A obesidade é uma doença multifatorial de alta prevalência que leva ao deenvolvimento de diversas comorbidades, representando desafios significativos para os sistemas de saúde. A cirurgia bariátrica e metabólica (CBM) consolidou-se como o tratamento mais eficaz para pacientes com obesidade; entretanto, no Brasil, o acesso limitado continua sendo uma barreira crítica.

**Objetivos:**

Avaliar as características clínicas, demográficas e metabólicas de pacientes com obesidade submetidos à CBM no Sistema Único de Saúde brasileiro e analisar a relação entre o tempo de espera e as comorbidades.

**Métodos:**

Foi realizado um estudo de coorte retrospectiva envolvendo 1.000 pacientes com obesidade subemtidos a tratamento entre julho de 2022 e junho de 2024. Variáveis clínicas, antropométricas e laboratoriais foram analisadas por meio de análise de regressão e testes estatísticos para avaliar a associação entre o tempo de espera e as comorbidades.

**Resultados:**

Foi encontrada correlação significativa entre maior tempo de espera e aumento do número de comorbidades (R²=0,686; p<0,001). Além disso, o número de comorbidades explicou 60% da variabilidade do tempo de espera (R²=0,600; p<0,001), com aumento médio de 1,92 anos para cada comorbidade adicional (IC95% 1,82–2,02). Pacientes que permaneceram na lista de espera por mais de 10 anos apresentaram maiores taxas de hipertensão arterial, diabetes mellitus tipo 2 e dislipidemia. O tempo de espera também impactou parâmetros da síndrome metabólica, incluindo hemoglobina glicada (Hb1Ac) (r=+680, p=0,031), lipoproteína de baixa densidade (LDL) (r=+640, p=0,044) e colesterol total (r=+830, p=0,008).

**Conclusões:**

O tempo prolongado de espera para a CBM está associado a uma maior carga de comorbidades metabólicas e às suas consequências.

## INTRODUCTION

 Obesity is a global public health problem affecting millions of people^11^. In Brazil, almost 80% of the population is overweight, with 22.3% suffering from obesity, contributing to an alarming rise in the prevalence of chronic diseases such as hypertension, type 2 diabetes mellitus (T2D), and dyslipidemia^23^. 

 Bariatric and metabolic surgery (BMS) has been shown to be effective and safe in ameliorating comorbidities. The medical literature provides robust evidence supporting the association between bariatric surgery and reduced all-cause mortality, as well as increased life expectancy^3,5,20^. 

 Overall, the medical literature supports the notion that early intervention with BMS in patients with obesity can lead to improved cardiovascular outcomes, including reduced mortality and incidence of cardiovascular events^22^. BMS has been shown to have renoprotective effects, potentially helping to prevent renal failure, and can significantly reduce the risk of progression from chronic kidney disease to kidney failure. BMS is associated with a 49.2% reduction in the hazard rate of death compared to usual care, with a median increase in life expectancy of 6.1 years^1^. 

 In fact, every chronic disease requires early diagnosis and adequate treatment to achieve good outcomes. Delays can lead to a poor prognosis in these patients. Over the last few decades, limited access to BMS in the Brazilian public health system has been observed, especially among vulnerable groups. This study aimed to analyze the clinical and metabolic characteristics of these patients in the public health system and to evaluate how wait time for surgery affects the presence of comorbidities and their consequences. 

## METHODS

 An observational retrospective cohort study was conducted with patients with obesity who were on the public health system waiting list and underwent BMS between July 2022 to June 2024. Inclusion criteria were body mass index (BMI)>30 kg/m² with uncontrolled diabetes mellitus (glycated hemoglobin — HbA1c>7 despite maximum medical therapy); BMI>35 kg/m² with associated comorbidities (hypertension, dyslipidemia, sleep apnea, among others); or BMI>40 kg/m² regardless of comorbidities. Patients who underwent revisional bariatric surgery were excluded. 

 Anthropometric parameters (weight, height, BMI, and abdominal circumference), glucose levels, HbA1c, lipid profile (low density protein — LDL, high density protein — HDL, and triglycerides), presence of comorbidities, and their sequelae were analyzed. Statistical analysis: continuous variables were described as means, standard deviations, and medians. The Shapiro-Wilk and Kolmogorov-Smirnov tests were used to assess normality. Associations between wait time and comorbidities were analyzed using linear regressions and χ^2^ tests, employing IBM SPSS Statistics, with the significance level set at 5% (p=0.05). The study was approved by the Ethics Committee of Universidade Federal do Rio de Janeiro (number 76686923.9.0000.5257). 

## RESULTS

**Figure 1:**
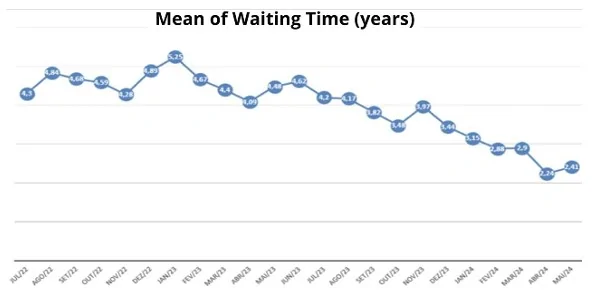
Waiting time for bariatric surgery. Evolution of the average waiting time, in years, for patients from July 2022 to May 2024.

**Figure 2:**
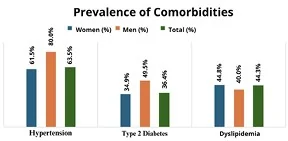
Prevalence of comorbidities in the study population stratified by gender.

 A total of 1,000 patients were included in the study: 89.6% were female and 10.4% were male, with a median age of 41.0 years. Of all patients, 19.6% had no comorbidities, 32.7% had one, 31.8% had two, and 15.9% had at least three comorbidities. Figure 1 shows the reduction in waiting time during the study period. Table 1 presents the baseline characteristics of the sample. The prevalence of comorbidities by gender is shown in Figure 2. 

**Table 1:** Patient characteristics.

Variable		N=1,000 (%)
Age, years		
	Median (SD)	41 (8.8)
	Min–Max	20–64
Gender, female		
	Percentage (%)	89.6
	Frequency	896
Waiting time, years		
	Median (SD)	4.0 (1.87)
	Min–Max	0.1–14
BMI, kg/m^2^		
	Median (SD)	46 (5.69)
	Min–Max	32.5–75.1
Abdominal circumference, cm		
	Median (SD)	127 (12.4)
	Min–Max	94–170
Fasting glucose, mg/dL		
	Median (SD)	106 (37.6)
	Min–Max	66–372
Hemoglobin A1c (HbA1c), %		
	Median (SD)	5.8 (1.2)
	Min–Max	4.5–12.5
LDL, mg/dL		
	Median (SD)	105 (35.7)
	Min–Max	88–270.8
HDL, mg/dL		
	Median (SD)	44.9 (19.7)
	Min–Max	31.1–65.4
Triglycerides, mg/dL		
	Median (SD)	128 (68.5)
	Min–Max	81.1–525
Total cholesterol, mg/dL		
	Median (SD)	178 (39.45)
	Min–Max	140.2–362

SD: standard deviation; BMI: body mass index; LDL: low density protein; HDL: high density protein.

**Figure 3:**
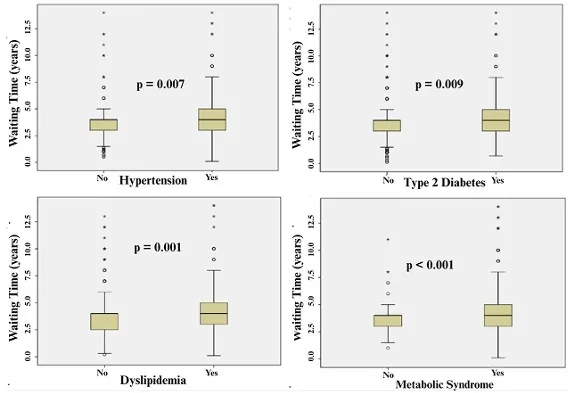
Waiting time until bariatric surgery according to the presence of comorbidities

 A significant association was observed between the number of comorbidities and waiting time (p<0.05). Patients with T2D, high blood pressure (HBP), dyslipidemia, and metabolic syndrome had longer waiting times until BMS (p=0.009, p=0.007, p=0.001, and p<0.001, respectively) (Figure 3). 

**Figure 4:**
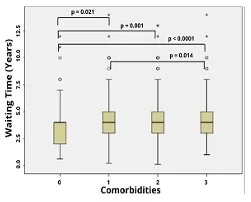
Number of comorbidities and waiting time until bariatric surgery.

 Significant differences in waiting time were observed among patients with different numbers of comorbidities (none *versus* one, p=0.021; none *versus* two, p=0.001; none *versus* three, p<0.001; one *versus* three, p=0.014) (Figure 4). 

 Significant differences were observed between patients with no comorbidities and those with one (p=0.021), two (p=0.001), and three comorbidities (p<0.0001). Additionally, patients with one comorbidity had shorter waiting times than those with three comorbidities (p=0.014). 

 Waiting time also had an impact on laboratory parameters associated with metabolic syndrome: Hb1Ac (r=+680, p=0.031); LDL (r=+640, p=0.044); and total cholesterol (r=+830, p=0.008). 

 Linear regression showed that the presence of comorbidities explained 68.6% of the variability in waiting time (R²=0.68; p<0.001), with an average increase of 3.39 years per comorbidity. Additionally, the number of comorbidities explained 60% of the variability in waiting time (R²=0.60; p<0.001), with an average increase of 1.92 years for each additional comorbidity (95%CI 1.82–2.02). 

 Fifty-two (5.2%) patients were admitted to the program with end-stage organ failure, including cirrhosis (n=17), major cerebrovascular events (n=15), heart failure (n=8), dialytic-dependent chronic kidney disease (n=7), lower-limb amputation (n=3), and blindness (n=2). All of these patients had waited more than eight years for bariatric surgery. 

## DISCUSSION

 Bariatric surgery is a well-established intervention for patients with severe obesity and metabolic syndrome, particularly for reducing cardiovascular risk. The timing of bariatric surgery should be considered based on several factors, including multidisciplinary evaluation and the patient’s physical and psychological readiness. However, it is imperative that patients are informed that longer wait times are associated with a higher risk of developing comorbidities and, consequently, increased perioperative risks. 

 The findings of this study emphasize the significant burden of metabolic comorbidities in patients with severe obesity awaiting BMS. The correlation between prolonged wait time and an increase in the number of comorbidities highlights a critical challenge for healthcare systems. 

 The high prevalence of hypertension (63.5%), T2D (36.4%), and dyslipidemia (44.3%) in this population notably exceeds that reported for the general Brazilian population in VIGITEL 2023 data^23^. These results are consistent with previous studies emphasizing the association between untreated obesity and an elevated risk of metabolic comorbidities^10,12,17^. 

 Firstly, the study by Luo et al. highlights that both the age at onset of obesity and cumulative exposure to obesity (measured in obese-years) are critical factors in the development of T2D. The study found that an earlier onset of obesity and a greater number of obese-years were associated with a higher risk of developing diabetes^13^. Similarly, the CARDIA study by Reis et al. demonstrated that the duration of abdominal obesity, independent of the degree of adiposity, is associated with an increased risk of diabetes. Each additional year of abdominal obesity was linked to a 4% higher risk of developing diabetes, emphasizing the importance of preventing or delaying the onset of obesity^18^. According to the Framingham Heart Study, there is a positive association between the duration of obesity and incident hypertension, particularly in women. However, this association diminishes when adjusted for time-varying BMI, suggesting that the immediate level of adiposity may be more critical than the duration of obesity in influencing hypertension risk^21^. Additionally, the Multi-Ethnic Study of Atherosclerosis suggests that both the severity and duration of obesity are associated with a higher risk of developing metabolic syndrome, which includes hypertension as a component. This implies that prolonged exposure to obesity increases the risk of cardiometabolic diseases, including hypertension^14^. 

 The literature suggests that BMS is beneficial in reducing cardiovascular risk factors and improving outcomes in patients with obesity and metabolic syndrome^9,22^. In patients with T2D and obesity, BMS has been associated with a lower incidence of macrovascular events, including coronary artery disease and cerebrovascular events, compared to nonsurgical management^2,9^. In the present sample, regression analysis revealed that wait time was a determining factor in the severity of metabolic conditions. Patients who waited more than 10 years for surgery showed a significant increase in the prevalence of hypertension, diabetes, and dyslipidemia. These results align with international studies indicating that delays in access to bariatric surgery exacerbate disease burden and complicate clinical management^6,7,15^. A total of 52 (5.2%) patients had end-stage diseases, all of whom had waited more than eight years for surgery. 

 The timing of surgery is crucial, as earlier intervention may lead to greater improvements in cardiovascular risk factors. Studies have shown that younger patients and those who achieve more substantial weight loss after surgery experience greater reductions in cardiovascular risk factors^16^. Therefore, considering bariatric surgery earlier in the course of severe obesity and metabolic syndrome, particularly in younger patients, may optimize cardiovascular outcomes. 

 Gender appears to influence both the severity and incdence of obesity and metabolic syndrome, with notable differences observed between men and women. The prevalence of obesity is generally higher in women than in men, particularly in low- and middle-income countries, where women have 2.72-fold higher odds of obesity than men^19^. Differences in fat distribution have important implications for metabolic health, as abdominal obesity is associated with a higher risk of metabolic syndrome and cardiovascular diseases^4^. Women generally exhibit greater insulin sensitivity and secretory capacity, which may offer some protection against metabolic syndrome and T2D during their reproductive years. However, these advantages diminish as glucose tolerance deteriorates^19^. In contrast, men are more susceptible to the metabolic consequences of obesity, including insulin resistance and hyperglycemia, which may contribute to the higher obesity-associated mortality rates observed in men^24^. The greater female representation (89.6%) in this study reflects a trend reported in the literature, whereby women more frequently seek medical interventions for obesity management^8^. However, men demonstrated a more severe clinical profile at the time of surgery, characterized by higher prevalence rates of hypertension and diabetes, which may be attributable to delays in seeking medical care. 

 Public politics need to be aligned to reduce wait times for BMS. This approach is less costly and provides better outcomes for patients. Policies aimed at increasing care capacity, improving infrastructure, and prioritizing high-risk patients may mitigate the progression of comorbidities and reduce the costs associated with managing these conditions. 

## CONCLUSIONS

 Prolonged wait time for bariatric and metabolic surgery was associated with an increased number of comorbidities and their complications, particularly hypertension, T2D, and dyslipidemia. Similarly, laboratory parameters associated with metabolic syndrome, such as HbA1c, LDL, and total cholesterol, also worsened significantly with delayed bariatric surgery. 

## Data Availability

The datasets generated and/or analyzed during the current study are available from the corresponding author upon reasonable request. The information regarding the investigation, methodology, and data analysis of the article is archived under the responsibility of the authors.
